# Characterization of a Novel BTD Hypomorphic Variant in a Patient with Complex Neurodevelopmental Delay: Resolving Actionable Metabolic Vulnerabilities Beyond Borderline Plasma Biochemistry

**DOI:** 10.3390/ijms27156847

**Published:** 2026-07-30

**Authors:** Claudia Toledo-Pacheco, Minerva Montero-Hernández, María Pilar López-Garrido, Carles de Diego-Boguñá, Francisco Sánchez-Sánchez

**Affiliations:** 1 Servicio de Genética, Hospital Universitario de Toledo (HUT), 45007 Toledo, Spain; claudiat@sescam.jccm.es (C.T.-P.); cadedi@sescam.jccm.es (C.d.D.-B.); 2 Instituto de Biomedicina, Universidad de Castilla-La Mancha (UCLM), 02008 Albacete, Spainfrancisco.ssanchez@uclm.es (F.S.-S.); 3 Facultad de Medicina CR, Universidad de Castilla-La Mancha (UCLM), 13071 Ciudad Real, Spain; 4 Facultad de Medicina AB, Universidad de Castilla-La Mancha (UCLM), 02008 Albacete, Spain

**Keywords:** biotinidase deficiency, *BTD* gene, variant of uncertain significance (VUS), functional validation, hypomorphic mutations, diagnostic odyssey, precision medicine

## Abstract

Plasma biochemistry often presents significant limitations in diagnosing borderline metabolic disorders, particularly within complex neurodevelopmental phenotypes. Here, we present the clinical genomic evaluation of a six-year patient presenting with early-onset hypotonia and severe gastrointestinal complications whose newborn screening panel did not evaluate biotinidase (BTD) activity. While initial baseline plasma biochemistry yielded borderline residual BTD function (46% of the population mean), targeted sequencing identified a novel, compound heterozygous hypomorphic variant (p.Thr459Met) in *trans* with the common p.Asp424His allele. In vitro functional validation confirmed that p.Thr459Met induces severe protein misfolding and intracellular retention, impairing enzyme secretion. Biotin supplementation triggered a documented and favorable therapeutic improvement, establishing this borderline enzymatic background as an actionable metabolic vulnerability unmasked by chronic gastrointestinal stressors. This study underscores the critical value of functional genomic characterization over static enzymatic biomarkers to identify highly treatable metabolic components within heterogeneous clinical landscapes.

## 1. Introduction

Biotinidase (BTD) deficiency (OMIM #253260) is an autosomal recessive metabolic disorder characterized by the inability to recycle endogenous biotin (vitamin B7) or release it from dietary proteins. This disruption leads to secondary biotin depletion, causing a wide spectrum of clinical manifestations, including hypotonia, respiratory difficulties, hearing loss, seizures, and psychomotor delay. Fortunately, early biotin supplementation can alleviate and often completely prevent the onset of these symptoms, making timely diagnosis crucial [1,2].

Clinically, BTD deficiency is classified into two primary forms based on residual serum enzyme activity: profound and partial deficiency [3]. Individuals with profound untreated deficiency (serum BTD activity less than 10% of the normal mean) present with severe, early-onset clinical findings such as intractable seizures, profound hypotonia, eczematoid skin rashes, alopecia, ataxia, and severe developmental delay. If left untreated, life-threatening metabolic complications, including ketolactic acidosis, organic aciduria, and hyperammonemia, may arise. In contrast, individuals with partial BTD deficiency (10% to 30% of mean normal activity) typically present a milder phenotype and may even remain asymptomatic. However, during periods of metabolic stress—such as infection, fever, or fasting—they can decompensate and develop severe neurological and cutaneous symptoms analogous to profound deficiency [4]. Serum BTD activity levels greater than 30% are generally considered normal [5,6]. Notably, the clinical spectrum is broad; late-onset presentations have been documented in adults suffering from optic neuropathy and/or peripheral neuropathy, who were frequently misdiagnosed with multiple sclerosis before severe BTD deficiency was ultimately identified [7].

The disorder is driven by loss-of-function (LOF) pathogenic variants in the *BTD* gene (NCBI: 686; HGNC: 1122). These genetic alterations can result in either null alleles, causing complete absence of functional enzyme, or hypomorphic alleles, which produce enzymes with residual, partial activity. This allelic heterogeneity directly contributes to the vast phenotypic variability observed in patients [8]. One of the most prevalent hypomorphic variants is NP_001357587.1:p.(Asp424His) (often historically referred to in the literature as p.Asp444His) [9]. Compound heterozygosity for this hypomorphic variant in *trans* with a null BTD variant typically results in partial BTD deficiency, yielding an expected enzymatic activity of approximately 20–25% [9,10]. Conversely, individuals homozygous for the p.(Asp424His) variant generally retain 45–50% enzyme activity—similar to heterozygous carriers of a single null variant—and usually do not require biotin supplementation. Despite these established patterns, patients with atypical or borderline enzymatic activity often suffer misdiagnoses due to non-specific clinical features and inconclusive laboratory screenings.

While next-generation sequencing (NGS) has revolutionized the identification of rare metabolic disorders, the accumulation of variants of uncertain significance (VUS) frequently hinders conclusive clinical interventions. To address this bottleneck, we present a multidisciplinary translational workflow investigating a patient with an atypical clinical presentation of partial biotinidase (BTD) deficiency. Trio-exome analysis revealed that the proband harbored compound heterozygous mutations in the *BTD* gene, consisting of the known hypomorphic allele p.(Asp424His) and a novel VUS, p.(Thr459Met). This study aims to decipher the structural and functional consequences of the p.(Thr459Met) substitution. Through a comprehensive paradigm including in silico molecular modeling, in vitro expression mapping, and quantitative in vivo plasma enzymatic assays, we demonstrate the deleterious nature of this variant, enabling its formal clinical reclassification and supporting precision diagnosis in an atypical metabolic phenotype.

## 2. Results

### 2.1. Clinical Presentation and Diagnostic Odyssey

The patient is a six-year-old girl referred to the Clinical Genetics Department due to moderate developmental delay, gait alterations, feeding difficulties, and recurrent respiratory infections. She is the second child of a healthy, non-consanguineous couple. The pregnancy was achieved via in vitro fertilization (dichorionic, diamniotic twin pregnancy) following two years of primary infertility. Delivery was induced at 38 + 3 weeks, resulting in a cesarean section with no neonatal complications. Birth weight was 2630 g, and standard newborn metabolic and otoacoustic screenings were reported as normal (notably, BTD screening was not included in the national newborn screening program at that time).

The patient’s diagnostic odyssey commenced at two months of age when she was evaluated for hypotonia and early developmental delay. Early intervention and occupational therapy were initiated at four months. Throughout her early childhood, she suffered from frequent respiratory infections (bronchiolitis, pneumonia, pharyngitis) and gastrointestinal issues, including frequent vomiting and solid feeding intolerance. At six years of age, she lacks bladder control, exhibits a clumsy gait with frequent falls and poor protective reflexes, and requires educational support, physiotherapy, and speech therapy.

Extensive clinical evaluations yielded largely non-specific findings. Brain MRI, electroencephalogram (EEG), Brainstem Evoked Response Audiometry (BERA), echocardiography, and abdominal ultrasounds were normal. Ophthalmologic evaluation revealed pale optic discs, and Visual Evoked Potentials (VEP) detected mild-to-moderate cortical delay. Electromyography (EMG) indicated a mild myopathy, accompanied by slightly elevated transaminases and creatine kinase (CK).

Initial genetic testing, including chromosomal microarray analysis (CMA), was normal. Subsequent specific multi-gene panel targeted toward primary neurodevelopmental disorders, intellectual disability, and early-onset hypotonia identified three heterozygous variants: a VUS in *RLIM* (p.Gly74Ser), a VUS in *KMT2C* (p.Ser3588Leu), and a likely pathogenic variant in *ATR* (p.Ala669GlufsTer4) (Supplementary Table S1). *A priori*, none of these findings adequately explained the patient’s phenotype. Finally, an unpanelled Trio-Exome sequencing analysis was carried out at a different sequencing company (Table S1), revealing two variants in compound heterozygosis in exon 4 of the *BTD* gene: the well-established hypomorphic pathogenic variant NM_001370658.1:c.1270G>C; p.(Asp424His) and a novel VUS, NM_001370658.1:c.1376C>T; p.(Thr459Met). The latter has a minor allele frequency of 0.08% in gnomAD and had not been previously reported in BTD-deficient patients.

Following this genetic finding, serum BTD enzymatic activity was measured, revealing a level of 4.90 nmol/min/mL which sits significantly below the laboratory standard reference range (7.80–13.50 nmol/min/mL). Given that the median value of the normal reference range is 10.65 nmol/min/mL, the patient retains an in vivo residual enzymatic activity of 46.0% relative to the population median. These metrics places the proband within the upper boundaries of a partial biotinidase deficiency state (typically defined between 10–30% for classic screening but expanded up to 60% in fluctuating hypomorphic clinical phenotypes), shedding light on her susceptibility to secondary metabolic decompensation. She was subsequently started on oral biotin supplementation (10 mg/day). After 14 months of continuous therapy, a comprehensive multidisciplinary clinical re-evaluation documented notable progress across all evaluated developmental domains. In the motor domain, she exhibited a significant reduction in generalized hypotonia, a more stable and coordinated gait, and a marked reduction in frequent falls. In the cognitive and communication domains, improvements were observed in verbal expression, comprehension, and attention span during educational tasks. Furthermore, she demonstrated enhanced social-emotional behavior and increased personal autonomy, successfully achieving daytime bladder control. In addition, she has not experienced any seizures and shows no signs of neuropathy, ataxia or optic atrophy. However, despite these positive therapeutic responses, the patient’s developmental trajectory did not achieve full chronological normalization. At her current chronological age of 8 years, specialized neuropediatric evaluations estimate her overall cognitive and motor performance to be aligned with a developmental age of slightly under 6 years.

**Figure 1:**
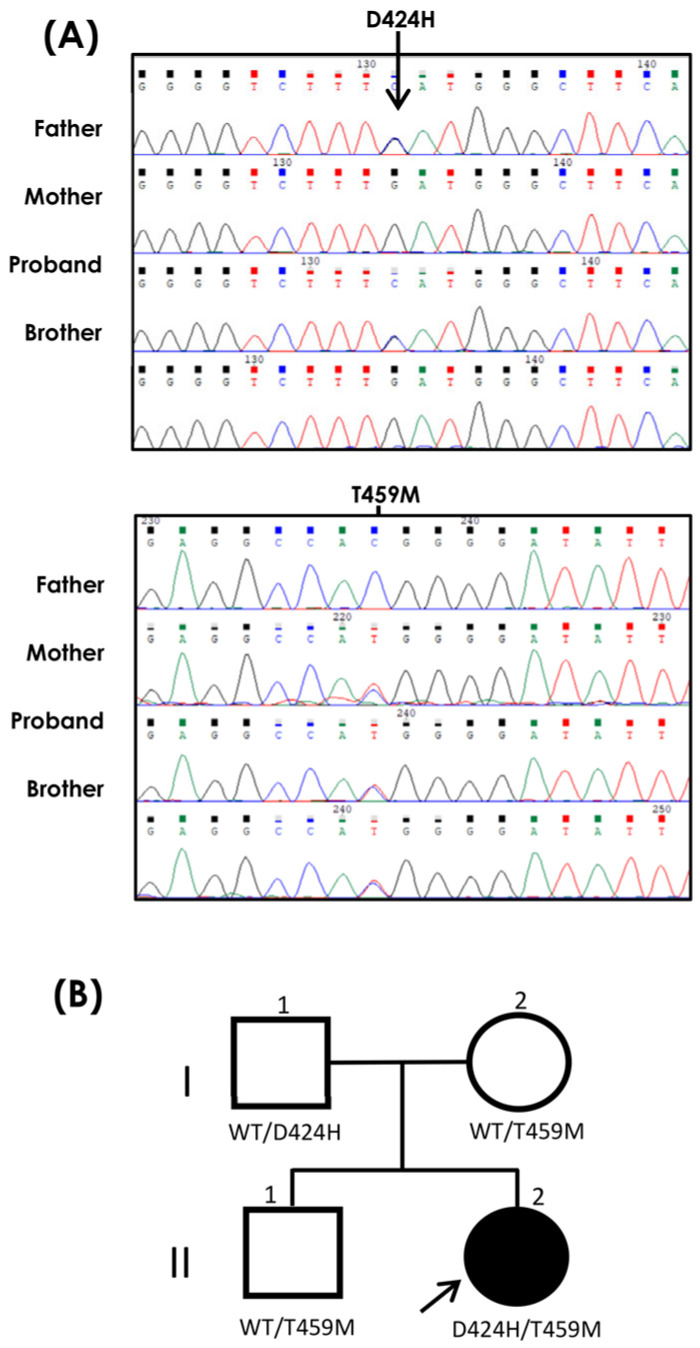
(**A**) Sanger confirmation of variants in the proband and family members. Arrows show the mutated nucleotide for each variant. (**B**) Genealogy of the family studied. Genotypes for both variants are shown below the symbols. WT: wild type allele. I or II indicates the generation.

Sanger sequencing successfully confirmed the compound heterozygous state in the proband and the carrier status in the unaffected parents and sibling (Figure 1), consistent with an autosomal recessive inheritance pattern.

### 2.2. In Silico and Structural Analysis of the BTD p.(Thr459Met) Variant

**Figure 2:**
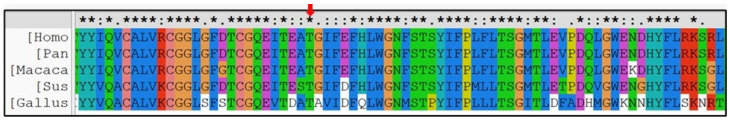
Multiple sequence alignment of BTD protein in various species on the evolutionary scale, including humans in the first row. Arrow indicates residue T459. Asterisk (*) indicates that at that position the residues are 100% identical. Two dots (:), indicate positions where conservative substitutions have been made. One dot (.), indicates positions where less conservative substitutions have been made.

To preliminarily assess the impact of the p.(Thr459Met) variant, computational predictive algorithms were employed. Although certain algorithms predicted the substitution to be tolerated, evolutionary conservation analysis demonstrated that the Thr459 residue is strictly conserved across multiple species, even in non-mammals, underscoring its functional or structural importance (Figure 2). Additionally, several missense variants in close proximity to this residue (e.g., p.Ala458Pro, p.Ala458Thr, p.Gly460Arg, p.His465Gln, and p.Leu466Gln) are established pathogenic mutations [11,12,13,14].

**Figure 3:**
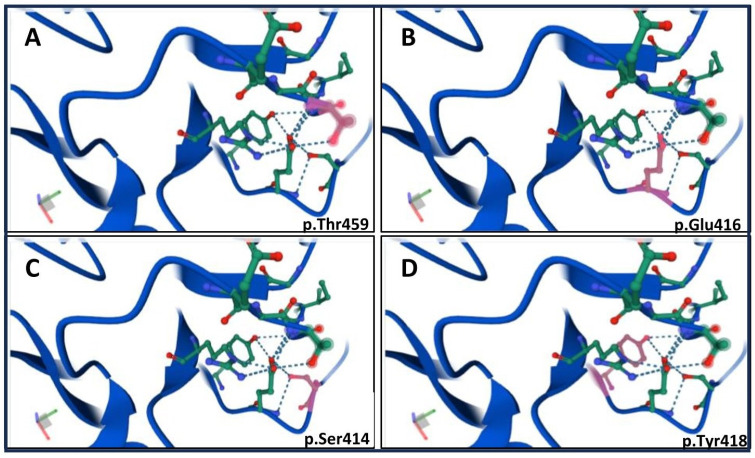
Three-dimensional structure of biotidinase showing the interactions of the Thr459 residue with 3 amino acids. In pink the affected amino acids are shown, Thr459 in (**A**), Glu416 in (**B**), Ser414 in (**C**) and Tyr418 in (**D**).

Three-dimensional in silico modeling further supported the deleterious nature of the substitution. The Thr459 residue directly interacts with Glu416, Ser414, and Tyr418 (Figure 3). Notably, mutations in Glu416 and Tyr418 have been previously reported in patients with BTD deficiency [15,16]. Although Thr459 does not interact directly with Ala419—a key residue for maintaining the regional three-dimensional structure—an amino acid change at position 459 could disrupt this intricate interaction network and critically destabilize the protein.

### 2.3. In Vitro Functional Characterization: Protein Expression and Enzymatic Activity

To definitively clarify the clinical significance of the p.(Thr459Met) variant, in vitro functional assays were performed. The known hypomorphic variant p.(Asp424His) [9], along with the established pathogenic variants p.(Gln436His), p.(Ala458Thr), and p.(Arg518Ser) [10,12,17], were included as positive controls for impaired function, while the p.(Pro371Ser) variant served as a benign control [10,12,17].

**Figure 4:**
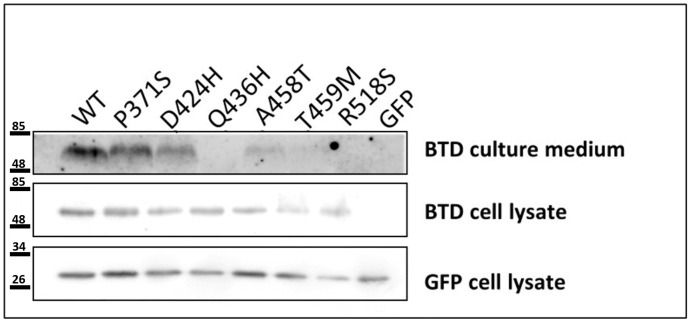
Biotidinase expression (wild type and mutant versions) in culture medium and cell lysate. Lanes 1–7 represent co-transfection profiles of BTD variants with a GFP reporter vector used as an internal transfection control. The final lane (labeled as GFP) represents a single transfection of the GFP vector alone, serving as a negative control.

Western blot analysis of transfected HEK-293T cells (Figure 4) revealed both intracellular and extracellular expression of the wild-type (WT) BTD, the benign p.(Pro371Ser) variant, and the hypomorphic p.(Asp424His) variant. However, the novel p.(Thr459Met) variant exhibited a dramatic reduction in expression, particularly in the extracellular culture medium, mirroring the behavior of severe pathogenic controls like p.(Gln436His) and p.(Arg518Ser), which were only detectable in the cell lysate. Densitometric quantification of the extracellular protein fractions confirmed this secretory impairment, revealing that the p.(Thr459Met) variant retains only 37.0% of the secretion efficiency observed in the WT BTD (Supplementary Figure S1).

**Figure 5:**
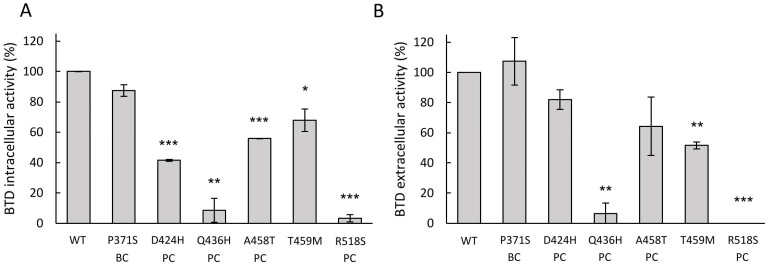
Biotinidase enzyme activity (wild type and mutant versions) in cell lysates (**A**) and in extracellular medium (**B**). BC: benign control; PC: pathogenic control. *: *p* < 0.05; **: *p* < 0.01; ***: *p* < 0.001.

Subsequent in vitro enzymatic assays corroborated these findings (Figure 5). All pathogenic controls exhibited significantly reduced enzymatic activity both intracellularly and extracellularly, whereas the benign control-maintained WT-like activity. The p.(Thr459Met) variant demonstrated significantly diminished biotinidase activity, falling strictly within the range of the pathogenic controls, confirming its deleterious impact on protein function and secretion.

### 2.4. In Vivo Validation and Variant Reclassification

**Figure 6:**
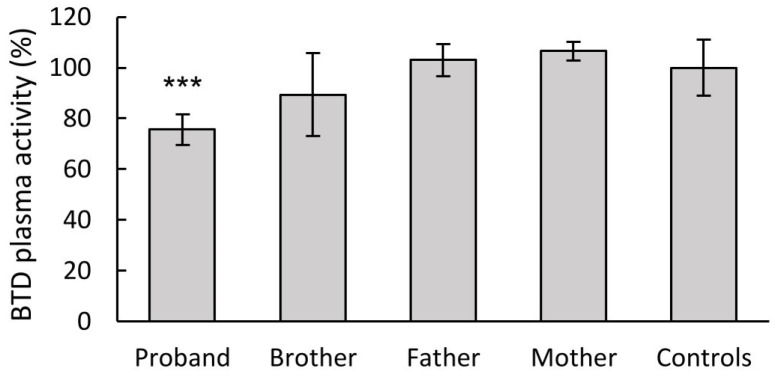
Biotinidase enzyme activity in the plasma of the proband and family members. The activity of the controls represents the average of 5 different individuals and the asymptomatic family members. ***: *p* < 0.001.

To correlate the in vitro findings with physiological conditions, in vivo BTD activity was quantified in plasma samples from the proband, asymptomatic family members, and five unrelated healthy controls (Figure 6). While unaffected family members and controls exhibited normal relative BTD activity (ranging from 89.4% to 106.6% of the normalized WT average), the proband displayed a significant reduction, retaining a localized relative activity of 75.8% (representing a 46.0% absolute reduction compared to the general population baseline).

In light of the compelling functional evidence (impaired protein secretion and reduced in vitro enzymatic activity) combined with the in vivo enzymatic data and family segregation, the p.(Thr459Met) variant is reclassified as “Likely Pathogenic” according to the ACMG criteria (PM2, PP3, PM3, PS3). It operates as a loss-of-function (LOF) hypomorphic variant which, in compound heterozygosity with the p.(Asp424His) variant, induces partial BTD deficiency.

## 3. Discussion

### 3.1. Functional Characterization and Molecular Impact of the Novel p.(Thr459Met) Variant

The advent of next-generation sequencing (NGS) has significantly accelerated the genetic identification of rare metabolic diseases; however, the frequent detection of variants of uncertain significance (VUS) remains a major bottleneck for definitive clinical management. In this study, we present a translational approach to characterize a complex clinical case with a prolonged pre-diagnostic period associated with a borderline partial biotinidase (BTD) deficiency. Trio-exome sequencing identified compound heterozygosity in the *BTD* gene involving the well-established hypomorphic pathogenic variant p.(Asp424His) and a novel missense VUS, p.(Thr459Met).

To reclassify p.(Thr459Met), in silico modeling and evolutionary conservation studies provided the first clues of its pathological nature. However, because computational predictions are not definitive, functional in vitro validation was paramount. BTD is known to be a secreted protein that exhibits both intracellular and extracellular expression [17], although the underlying mechanisms governing this duality remain poorly understood. Our in vitro results demonstrate that the p.(Thr459Met) variant behaves similarly to the established pathogenic control p.(Ala458Thr), exhibiting a drastic reduction in expression, particularly in the culture medium (37% relative to WT), which suggests a severe impairment in protein secretion.

Furthermore, we observed that pathogenic mutations exhibit distinct intracellular versus extracellular activity profiles, as previously reported [18]. The p.(Asp424His) variant showed the greatest discrepancy (41.5% intracellular vs. 81.9% extracellular activity), whereas the p.(Thr459Met) variant displayed reduced and variable activity (67.8% intracellular vs. 51.6% extracellular). It has been proposed that the deleterious effect of p.(Asp424His) is primarily related to a decrease in protein quantity rather than a complete loss of intrinsic catalytic activity [18], explaining its high phenotypic variability. In fact, neither the Asp424 nor the Thr459 residues belong to the core catalytic active site of the protein [19]. Based on our functional data, the genotype identified in the proband involves two hypomorphic variants in *trans*, where p.(Thr459Met) exerts a more severe loss-of-function effect than p.(Asp424His), leading to a mild reduction in overall BTD activity.

### 3.2. Deciphering the Biochemical Discrepancies and In Vivo Kinetics

A notable finding in our study was the discrepancy between the patient’s severe in vitro enzymatic and secretion impairment of p.(Thr459Met) variant and her in vivo relative plasma BTD activity, which stood at 75.8% (with an absolute population-level reduction of 46.0%). Classically, partial BTD deficiency is biochemically defined by a residual serum enzyme activity between 10% and 30% of the mean normal control value. While the proband’s activity falls above this traditional screening threshold, her absolute values were consistently below the standard laboratory reference ranges, placing the patient in the upper boundaries of partial BTD deficiency.

This borderline biochemical phenotype can be robustly attributed to the specific genotypic combination identified. The p.(Asp424His) variant is notorious for its high residual activity, partial structural stability, and structural proneness to accelerated thermal inactivation, which frequently leads to borderline or fluctuating total serum BTD measurements [9]. Indeed, a thorough review of the literature highlights a significant discrepancy in the genotype–phenotype correlation among individuals sharing hypomorphic backgrounds (Table 1).

As compiled in Table 1, identical compound heterozygous or homozygous hypomorphic genotypes can result in clinical outcomes ranging from completely asymptomatic individuals to patients presenting with profound biotinidase deficiency. This phenotypic plasticity demonstrates that a residual enzyme activity does not act as a binary diagnostic switch. Instead, it establishes a constitutional metabolic vulnerability where other genetic, epigenetic, or microenvironmental factors could contribute to the ultimate clinical presentation. It should be noted, furthermore, that in these examples, the absolute biotinidase activity is even lower than that detected in the present case (4.90 nmol/min/mL), suggesting that herein the modifying factors may be even more significant.

Beyond these assay kinetics, it is well-documented that clinical manifestations and BTD activity can be further influenced by preanalytical variables—such as age, prematurity, neonatal jaundice, and sample temperature during storage or transport [22,23,24,25] as well variable dietary biotin intake. Indeed, while most individuals with partial BTD deficiency [26] and even some with profound deficiency [21] remain asymptomatic, up to 28% of patients suffer from delayed diagnoses [26], underscoring that current standard detection methods may be insufficient for mild or atypical presentations.

On the other hand, it must be acknowledged that these enzymatic metrics represent single-point determinations. While longitudinal, multi-day sampling would provide a mathematically definitive kinetic average, the immediate recovery of the pediatric patient upon starting biotin supplementation rendered further repetitive, invasive blood draws ethically untenable. This overlapping, borderline enzymatic behavior mirrors other metabolic conditions involving hypomorphic backgrounds, such as the p.Asn370Ser variant in Gaucher disease (*GBA1*) [27,28] or mild *ACADM* mutations in MCAD deficiency [29,30], where laboratory values frequently fluctuate across established diagnostic thresholds.

### 3.3. A Hypothetical Multifactorial Model for the Clinical Phenotype

Given the functional proof that p.(Thr459Met) severely restricts protein secretion and activity, the patient’s overall biochemical status is best described as a state of significantly reduced, borderline BTD activity. Rather than a classic metabolic block, this genetic background could create a vulnerability to secondary biotin depletion. Symptomatic partial BTD deficiency is exceedingly rare and typically manifests much later in life under intense physiological stress. Therefore, the proband’s severe symptoms at two months of age—alongside normal organic acid and acylcarnitine profiles—suggest that reduced BTD activity alone may not be sufficient to explain the full multisystemic picture.

Instead, a plausible pathophysiological model may be built upon a multifactorial vulnerability, in which a secondary, diet-induced biotin deficiency could have compounded a constitutionally reduced enzymatic capacity. The patient’s chronic gastrointestinal manifestations, including persistent vomiting and severe solid feeding intolerance during early childhood, may have compromised systemic biotin availability over a prolonged period. When this environmental substrate restriction met a 46% residual enzymatic function, a metabolic threshold could have been crossed, potentially inducing a secondary functional restriction of holocarboxylase synthetase (HLCS) activity despite the absence of primary mutations in the *HLCS* gene. Crossing this threshold may have triggered the clinical symptoms that later responded to biotin therapy. Ultimately, this case illustrates that clinical diagnostic criteria should integrate functional genomic data alongside strict biochemical cutoffs, as individuals with borderline activity can still experience metabolic stress and derive benefit from biotin supplementation.

### 3.4. The Broader Clinical Landscape: Coexisting Factors and Future Diagnostic Directions

Crucially, the identification of the compound heterozygous *BTD* variants provided an immediate, highly actionable therapeutic target that improved the patient’s quality of life. However, her overall developmental delay is estimated to be approximately two years. This persistent deficit may, in part, be related to the late initiation of biotin therapy due to her prolonged diagnostic odyssey. This observation suggests that while late intervention can effectively rescue the active metabolic overlay, it may not completely reverse pre-existing neurodevelopmental delays accrued during critical early developmental windows.

In addition, the extreme precocity of her neurological presentation at two months of age suggests the potential coexistence of a parallel or synergistic primary neurodevelopmental etiology. In this context, it is noteworthy that the pregnancy was achieved via in vitro fertilization (IVF). Assisted reproductive technologies have been associated with an increased statistical risk of epigenetic modifications, specifically genomic imprinting disorders and DNA methylation alterations, which can lead to complex neurodevelopmental phenotypes [31,32,33]. Furthermore, prior single clinical exome screening highlighted rare variants of uncertain significance in chromatin remodeling and neural differentiation genes, such as *KMT2C* and *RLIM*, further pointing toward a heterogeneous genomic background.

Consequently, we hypothesize that the borderline BTD activity may act as a major compounding metabolic modifier rather than the sole driver of the phenotype. While the metabolic stabilization achieved with biotin supplementation represents a major clinical milestone, other underlying genetic or epigenetic factors cannot be definitively excluded. We recommend that the patient’s clinical management includes a periodic reassessment over the coming years, prioritizing advanced diagnostic modalities such as Whole Genome Sequencing (WGS) to capture non-coding regulatory variants, alongside comprehensive epigenetic/methylation profiling to investigate potential post-IVF imprinting defects.

In summary, our study illustrates that combining functional assays with genomic analysis is essential to overcome the limitations of standard biochemical analysis alone. It highlights that integrating plasma enzymatic assay with comprehensive genetic characterization is necessary to advance precision medicine in complex metabolic and neurodevelopmental disorders.

## 4. Materials and Methods

### 4.1. Genetic Diagnostic Workflow and NGS Methodology

#### 4.1.1. Sequential NGS Strategy

The patient underwent a sequential genetic diagnostic workflow. Initially, a clinical Whole Exome Sequencing (WES) focused on neurodevelopmental and hypotonia gene panels (targeted virtual panel) was performed using an Illumina NextSeq 500 platform (Eurofins Megalab, Madrid, Spain). This first test highlighted rare variants of uncertain significance (VUS) in *RLIM* and *KMT2C*, and a likely pathogenic variant in *ATR*, but lacked an unequivocal diagnostic match. Because the *BTD* gene was absent from this disease-specific panel design, potential alterations were not clinically prioritized or reported during this first analytical phase.

To further elucidate the etiology, a subsequent, independent *trio*-based Whole Exome Sequencing (*trio*-WES) was executed using the ExoNIM^®^ Trio platform (NIMGenetics, Madrid, Spain). This independent sequencing run successfully identified and confirmed the compound heterozygous variants in the *BTD* gene as well as secondary VUS in *TTN* and *LAMA2* genes.

The supplementary table sets out the list of genomic variants identified in the patient through sequential NGS studies, along with their clinical relevance.

#### 4.1.2. Trio-WES Platform and Target Capture

For the definitive *trio*-WES analysis, target enrichment and library preparation were conducted utilizing the Comprehensive Exome Panel (Twist Bioscience, San Francisco, CA, USA), which targets a 41.2 Mb region encompassing >20,000 genes, achieving >99% coverage of RefSeq, CCDS, and GENCODE databases. Massively parallel sequencing was executed on an Illumina NovaSeq 6000 System™. The sequencing run yielded raw outputs of 6.998 Gb for the proband, 7.442 Gb for the father, and 6.610 Gb for the mother.

#### 4.1.3. Alignment and Quality Metrics

Raw sequencing reads from the *trio*-WES were aligned against the human reference genome GRCh37/hg19 using the DRAGEN™ BioIT Platform (Illumina, version 07.021.510.3.5.7) for variant calling within the target regions (Twist_Exome_RefSeq_targets_hg19.bed).

The mean read depth achieved was 103x for the proband, 112x for the father, and 100X for the mother. The fraction of targets covered at ≥10x was 98.3% (proband), 98.4% (father), and 98.2% (mother). Targets covered at ≥20x reached 98.0% for both the proband and mother, and 98.1% for the father. The *BTD* gene achieved 100% coverage at a minimum depth of 20X across all family members.

#### 4.1.4. Variant-Specific Metrics

The specific alignment and allele metrics for the compound heterozygous *BTD* variants (transcript NM_000060.4) identified in Exon 4 were as follows (variant nomenclature has been standardized according to HGVS guidelines to reflect the mature peptide numbering, correcting the raw annotations from the clinical report). c.1270G>C; p.(Asp424His): Inherited from the father (heterozygous in both). The proband exhibited a read depth of 109x with a Variant Allele Frequency (VAF/Allele Balance) of 52%. The father showed a depth of 114x (VAF: 52%), and the mother showed a depth of 124x. c.1376C>T; p.(Thr459Met): Inherited from the mother (heterozygous in both). The proband exhibited a read depth of 88x with a VAF of 51%. The mother showed a depth of 105x (VAF: 56%), and the father showed a depth of 125x.

#### 4.1.5. Filtering Strategy and Variant Interpretation Workflow

Variant annotation was carried out using a proprietary pipeline integrating internal databases and open-access resources. The filtering workflow was restricted to exonic and splicing regions (±5 bp) with a minimum VAF threshold of >30%. Variants with a population frequency ≥1% in public databases—including gnomAD, dbSNP, 1000 Genomes Project, and NHLBI-ESP—or classified as benign/likely benign were filtered out.

For missense variants, in silico pathogenicity predictions were estimated using CADD scores and a combination of predictors from dbNSFP (including SIFT, PolyPhen2, MutationTaster, MutationAssessor, LRT, FATHMM, and MetaSVM). Nucleotide conservation was evaluated using PhyloP scores from UCSC. Copy Number Variation (CNV) screening was concurrently performed utilizing DRAGEN v4.4 software parameters against control samples. Phenotypic prioritization was conducted using a machine-learning-based pipeline integrated into the internal workflow. Final variant nomenclature and classification were determined following the guidelines of the Human Genome Variation Society (HGVS) and the American College of Medical Genetics and Genomics (ACMG).

### 4.2. Patient and Family Recruitment

The proband and her immediate family members (father, mother, and sibling) were recruited through the Clinical Genetics Department at the Hospital General Universitario de Toledo (Spain). A comprehensive medical examination was conducted, and serum biotinidase concentrations were independently determined by an external laboratory according to the protocol described by González et al. [34]. For the present genetic and functional studies, 5–10 mL of peripheral venous blood was collected from each participant following written informed consent. Samples were aliquoted and stored at −80 °C at the Medical Genetics Laboratory of the Universidad de Castilla-La Mancha (UCLM). The study was conducted in accordance with the Declaration of Helsinki. Formal ethical review and approval were waived for this specific study as the genetic and functional analyses were conducted within the scope of routine clinical diagnostic assistance under an official institutional agreement between the regional public health service (SESCAM) and the University of Castilla-La Mancha (UCLM) (code: CGP180358, date: 15 November 2018), rather than as part of a prospective research trial. Informed consent was obtained from all subjects involved in the study. Written informed consent has been obtained from the patient’s parents to publish this paper.

### 4.3. Computational Studies and Pathogenicity Prediction

To predict the potential pathogenicity of the identified genetic variants, multiple in silico prediction algorithms were utilized, including SIFT, PolyPhen-2, and Align GVGD. Tertiary protein structure predictions and interaction mappings were conducted using the AlphaFold Protein Structure Database (#AF-P43251-F1-v4; EMBL-EBI). Evolutionary conservation of the BTD protein amino acid residues was analyzed using ClustalX 2.1 software. The target residues (Asp424 and Thr459) were compared against known pathogenic control variants (Gln436, Ala458, Arg518) and a benign variant (Pro371) across diverse species along the evolutionary scale (*Homo sapiens*, *Pan troglodytes*, *Macaca mulatta*, *Sus scrofa*, and *Gallus gallus*).

### 4.4. DNA Extraction and Variant Confirmation

Genomic DNA (gDNA) was extracted from peripheral blood leukocytes using the E.Z.N.A. Blood DNA Kit (Omega Bio-tek, Norcross, GA, USA) following the manufacturer’s instructions. DNA concentration and purity were assessed using a NanoDrop ND-1000 spectrophotometer (Thermo Fisher Scientific, Waltham, MA, USA).

The genetic variants identified by NGS were validated in the proband and analyzed for segregation in the family members via Sanger sequencing. PCR amplification of the target genomic region was performed using existing primers originally designed for site-directed mutagenesis (BTD-P371S-UP and BTD-R518S-DW; Table 2). Although these primers are longer than standard sequencing oligonucleotides, they flanked the region of interest effectively, yielding a clean amplicon and high-quality sequencing reads without non-specific background. The reaction was performed in a 50 µL reaction volume containing 50 ng of gDNA template, 1X standard reaction buffer, 0.2 mM dNTP mixture, 0.2 µM of each primer, 2.5% DMSO, and 1 U of Taq DNA polymerase (Biotools, Madrid, Spain). Thermocycling conditions consisted of an initial denaturation at 94 °C for 5 min, followed by 40 cycles of 94 °C for 30 s, 55 °C for 20 s, and 72 °C for 30 s, with a final extension at 72 °C for 5 min. Terminator cycle sequencing was performed using the BigDye Terminator v3.1 Cycle Sequencing Kit (Applied Biosystems, Foster City, CA, USA), and products were analyzed on a 3730 xl Automated DNA Analyzer (Applied Biosystems).

### 4.5. Plasmid Constructs and Site-Directed Mutagenesis

To generate mutant clones, the variants of interest were introduced into a pCMV6 expression vector (OriGene, RC204153) encoding myc-DDK-tagged human biotinidase. Mutagenesis was performed using the QuikChange II XL Site-Directed Mutagenesis Kit (Agilent Technologies, Santa Clara, CA, USA) along with specific custom-designed primers (Table 2). Competent cells were transformed via heat shock with the recombinant DNA and grown on LB-agar plates supplemented with 50 µg/mL kanamycin. Positive clones were selected and confirmed by PCR and Sanger sequencing. Recombinant plasmids were purified using the GeneJET Plasmid Miniprep Kit (Thermo Fisher Scientific).

### 4.6. Cell Culture and Transient Transfection

HEK-293T cells were cultured in Dulbecco’s Modified Eagle Medium (DMEM, 1X) supplemented with 4.5 g/L glucose, L-glutamine, sodium pyruvate, 10% fetal bovine serum (Capricorn Scientific, Ebsdorfergrund, Germany), and 1% penicillin-streptomycin (Gibco, Grand Island, NY, USA). Transfections were performed in triplicate in 6-well plates using 1 µg of the respective mutant or wild-type (WT) plasmid and the Canfast transfection reagent (Canvax, Córdoba, Spain). For internal normalization and transfection efficiency control, a vector encoding a Green Fluorescent Protein (GFP) reporter gene was co-transfected alongside each BTD construct. Additionally, a single transfection with the empty GFP vector alone (without any BTD plasmid) was included to serve as a negative control for expression and antibody specificity. Cells and culture media were harvested 48 h post-transfection and stored at −80 °C.

### 4.7. Protein Detection by Western Blot

Thirty microliters of cell lysate or culture medium were denatured in Laemmli buffer at 95 °C for 5 min. Proteins were separated by SDS-PAGE (4% stacking gel, 10% resolving gel) at 30 mA for 2 h and subsequently transferred to a nitrocellulose membrane (Amersham, UK) using a semi-dry transfer system at 15 V for 10 min (Thermo Fisher Scientific). Membranes were blocked with 10% non-fat dry milk in TTBS for 1 h at room temperature on a shaker. Recombinant BTD protein was detected using a primary monoclonal anti-myc antibody (Santa Cruz Biotechnology, Dallas, TX, USA; diluted 1:500) incubated overnight at 4 °C. Following three washes, the membranes were incubated with an HRP-conjugated secondary IgG antibody (Santa Cruz Biotechnology; diluted 1:1000) for 1 h. Chemiluminescent signals were developed using the SuperSignal West Femto Maximum Sensitivity Substrate (Thermo Fisher Scientific) and visualized on an ImageQuant LAS 4000 imaging system. Densitometric quantification of the protein bands was performed using ImageJ 1.53e software (NIH, Bethesda, MD, USA). To evaluate secretion efficiency without the confounding bias of intracellular variations, the percentage of secretion for each variant was calculated as the ratio of extracellular signal intensity to total expressed protein (intracellular + extracellular signal), with the WT secretion set as 100% reference.

### 4.8. In Vitro BTD Enzymatic Assay

Harvested cells were lysed in 150 µL of RIPA buffer (Thermo Fisher Scientific) supplemented with protease inhibitors and DTT. Lysates were incubated on ice for 10 min, vortexed for 30 s twice, and centrifuged to clear cellular debris. To measure BTD enzymatic activity, 15 µL of either cell lysate or culture medium was mixed with 150 µL of substrate solution (0.15 M potassium phosphate pH 6.5, 0.1 mM DTT, and 50 µM biotinyl-6-aminoquinoline [BAQ]) and incubated for 3 h at 37 °C. The fluorescent product was quantified using spectrophotometric techniques with an excitation wavelength of 350 nm and an emission wavelength of 520 nm.

### 4.9. In Vivo BTD Enzymatic Assay

Plasma was isolated from 4 mL of peripheral blood collected from all family members and five unrelated healthy control individuals (without symptoms of BTD deficiency). Blood was centrifuged at 2000× *g* for 10 min. Plasma aliquots of 500 µL were immediately frozen at −80 °C until further use. The in vivo BTD enzymatic assay was performed using 15 µL of plasma, following the exact same protocol and substrate conditions described above for the in vitro assay.

### 4.10. Statistical Analysis

For recombinant BTD analyses, two independent in vitro experiments were conducted, each comprising triplicate measurements per variant. Enzymatic activity was expressed as a relative percentage of the WT BTD activity (defined as 100%) after subtracting the basal endogenous activity measured in the GFP-transfected negative control. For plasma in vivo BTD assays, triplicate measurements were obtained for each subject. Activity was expressed relative to the mean value of the healthy controls and asymptomatic family members (defined as 100%). Data are presented as the mean ± standard deviation (SD). Statistical significance was determined using a two-sample *t*-test (assuming equal variances) using Microsoft Excel (Microsoft 365 version). A *p*-value < 0.05 was considered statistically significant (*: *p* < 0.05; **: *p* < 0.01; ***: *p* < 0.001).

## 5. Conclusions

This case underscores the clinical utility of integrating targeted functional validation to clarify the impact of novel VUS and identify treatable metabolic factors within complex clinical presentations. Through molecular and biochemical characterization, we demonstrated the functional relevance of the novel p.Thr459Met variant, which exhibits a severe secretion defect and loss of enzymatic activity in vitro. When present in *trans* with the common hypomorphic p.Asp424His allele, this genetic background resulted in a state of significantly reduced, borderline BTD activity in vivo. Although the patient’s plasma activity sits above the classic 30% threshold traditionally associated with partial biotinidase deficiency, and while this metabolic trait may not fully account for the entirety of her complex early neurodevelopmental presentation, it represented a critical, treatable factor. We hypothesize that a secondary dietary substrate restriction—potentially compounded by chronic gastrointestinal symptoms—may have contributed to the clinical onset, rendering biotin supplementation effective in achieving multi-domain clinical stabilization.

Ultimately, this study illustrates that when standard biochemical tests yield borderline or inconsistent results, integrating plasma enzymatic activity profiling, genomic sequencing, and targeted molecular functional studies support timely diagnostic clarification and advance precision medicine in complex neurodevelopmental disorders.

## Figures and Tables

**Table 1:** Genotype–phenotype discrepancies and enzyme activity in biotinidase deficiency. * stop codon.

Genotype (Protein Change)	Phenotype	Biotinidase Activity	Reference
p.(Asp424His) + p.(Cys13Phefs*36)	Partial	1.45 nmol/min/mL	[20]
p.(Asp424His) + p.(Cys13Phefs*36)	Profound	0.11 nmol/min/mL	[20]
p.(Asp424His) + p.(Arg137His)	Profound	0.66 nmol/min/mL	[20]
p.(Asp424His) + p.(Arg137His)	Partial	1.83 nmol/min/mL	[20]
p.(Asp424His) + p.(Ala151Thr)	Asymptomatic	<1% of mean normal serum activity	[21]
p.(Asp424His) + p.(Ala151Thr)	Partial	1.68 nmol/min/mL	[20]
p.(Asp424His) + p.(Ala151Thr)	Profound	0.76 nmol/min/mL	[20]
p.(Asp424His) + p.(Asp424His)	Profound	0.48 nmol/min/mL	[20]
p.(Asp424His) + p.(Asp424His)	Partial	2.27 nmol/min/mL	[20]
p.(Asp424His) + p.(Thr512Met)	Partial	1.92 nmol/min/mL	[20]
p.(Asp424His) + p.(Thr512Met)	Profound	0.71 nmol/min/mL	[20]
p.(Cys13Phefs*36) + p.(Cys13Phefs*36)	Partial	2.39 nmol/min/mL	[20]
p.(Cys13Phefs*36) + p.(Cys13Phefs*36)	Profound	0.11 nmol/min/mL	[20]
p.(Arg137His) + p.(Arg137His)	Partial	2.60 nmol/min/mL	[20]
p.(Arg137His) + p.(Arg137His)	Profound	0.27 nmol/min/mL	[20]
p.(Asp232Gly) + p.(Asp232Gly)	Asymptomatic	1.5% of mean normal serum activity	[21]

**Table 2:** Primers used for targeted site-directed mutagenesis. Bold nucleotides indicate the introduced nucleotide changes.

Name	Primer (5′→3′ Sense)
BTD-P371S-UP	CAAGTGGAACGTGAATGCT**T**CTCCCACATTTCACTCTG
BTD-P371S-DW	CAGAGTGAAATGTGGGAG**A**AGCATTCACGTTCCACTTG
BTD-D424H-UP	GCCCTGGGGGTCTTT**C**ATGGGCTTCACACAG
BTD-D424H-DW	CTGTGTGAAGCCCAT**G**AAAGACCCCCAGGGC
BTD-Q436H-UP	GGCACTTACTACATCCA**C**GTGTGTGCCCTGGTC
BTD-Q436H-DW	GACCAGGGCACACAC**G**TGGATGTGTAAGTGCC
BTD-A458T-UP	GGACAGGAAATCACAGAG**A**CCACGGGGATATTTGAGT
BTD-A458T-DW	ACTCAAATATCCCCGTGG**T**CTCTGTGATTTCCTTGTCC
BTD-T459M-UP	CAGGAAATCACAGAGGCCA**T**GGGGATATTTGAGTTTCAC
BTD-T459M-DW	GTGAAACTCAAATATCCCC**A**TGGCCTCTGTGATTTCCTG
BTD-R518S-UP	GGCGGCTCTCTATGGG**T**GCTTGTATGAGAGGG
BTD-R518S-DW	CCCTCTCATACAAGC**A**CCCATAGAGAGCCGCC

## Data Availability

The original contributions presented in the study are included in the article. The p.(Asp424His) and p.(Thr459Met) BTD variants identified in the patient have been submitted to the Leiden Open Variation Database (LOVD) under the ID #0001047048. Further inquiries can be directed to the corresponding author.

## Supplementary Materials

The following supporting information can be downloaded at https://www.mdpi.com/article/10.3390/ijms27156847/s1.

## Abbreviations

The following abbreviations are used in this manuscript:

NGS	Next-generation sequencing
BTD	Biotinidase
VUS	Variant of uncertain significance
LOF	Loss of function
