# Supplementary material for: Characterization of a Novel BTD Hypomorphic Variant in a Patient with Complex Neurodevelopmental Delay: Resolving Actionable Metabolic Vulnerabilities Beyond Borderline Plasma Biochemistry
Source: Int J Mol Sci. 2026 Jul 30;27(15):6847. doi: 10.3390/ijms27156847 (PMC13467485; doi:10.3390/ijms27156847)
Supplement: Supplementary file 1 [file ijms-27-06847-s001.zip › ijms-4374651-supplementary.pdf]

Supplementary Table S1. List of rare genomic variants identified in the proband across sequential NGS studies.

Study/Platform	Gene (HGNC)	HGVS cDNA	HGVS Protein	Zygosity	Inheritance	ACMG Classification	Clinical Relevance/ Phenotype
Gene panel◊	*KMT2C*	c.3412G>A	p.(Val1138Ile)	Heterozygous	NA	VUS	Chromatin remodeling; potential neurodevelopmental co-etiology.
Gene panel◊	*RLIM*	c.182A>G	p.(Asn61Ser)	Heterozygous	NA	VUS	Neural differentiation; potential synergistic genetic background.
Gene panel◊	*ATR*	c.2006_2007delCT	p.(Ala669Glufs*4)	Heterozygous	NA	Likely Pathogenic	Cutaneous telangiectasia and oropharyngeal cancer (#614564)
Trio-Exome	*BTD*	c.1270G>A	p.(Asp424His)	Heterozygous	Maternal	Pathogenic (hypomorphic)	Partial biotinidase deficiency; compounding metabolic modifier.
Trio-Exome	*BTD*	c.1376C>T	p.(Thr459Met)	Heterozygous	Paternal	VUS (reclassified in the present work as Likely Pathogenic)	Novel variant characterized in this study; restricts protein secretion.
Trio-Exome	*TTN*	c.82904A>G	p.(Asn27635Ser)	Heterozygous	Paternal	VUS	Giant muscle protein; unlikely to contribute isolation to the phenotype but monitored for neuromuscular background.
Trio-Exome	*LAMA2*	c.4451G>A	p.(Arg1484His)	Heterozygous	Maternal	VUS	Merosin-deficient congenital muscular dystrophy background; potential modifier for early hypotonia.

◊ Gene panel associated with neurodevelopmental disorders and hypotonia. NA: Not available; VUS: Variant of Uncertain SignificanceVUS: Variant of Uncertain Significance. The truncating variant in *ATR* in heterozygous state is only linked to a familial risk of adult-onset telangiectasia/neoplasia, ruling out autosomal recessive Seckel syndrome in the proband.

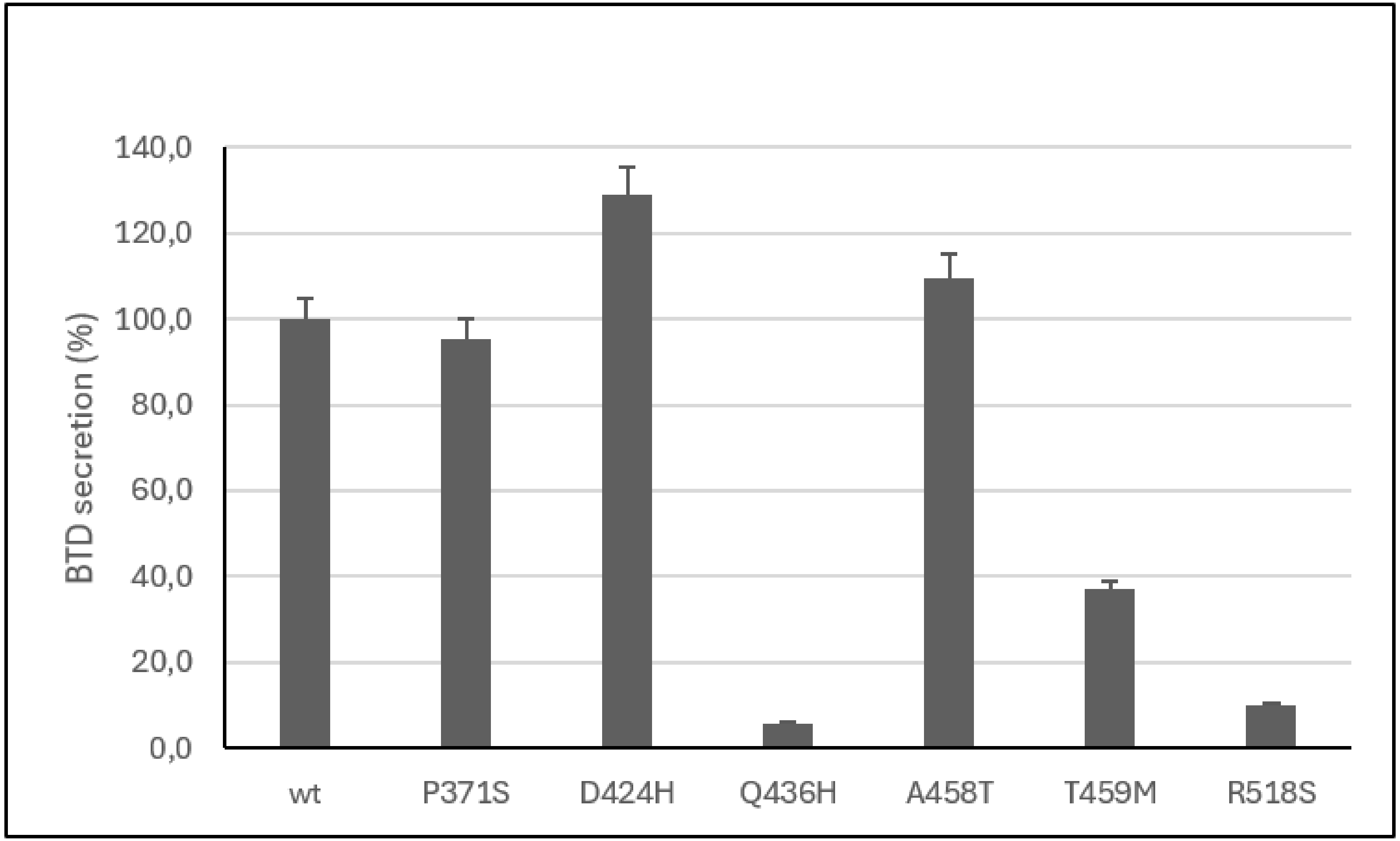

Supplementary Figure S1. Percentage of secretion by different BTD mutants and the wild-type recombinant proteins.
